# Molecular and Functional Study of Transient Receptor Potential Vanilloid 1-4 at the Rat and Human Blood–Brain Barrier Reveals Interspecies Differences

**DOI:** 10.3389/fcell.2020.578514

**Published:** 2020-11-11

**Authors:** Huilong Luo, Bruno Saubamea, Stéphanie Chasseigneaux, Véronique Cochois, Maria Smirnova, Fabienne Glacial, Nicolas Perrière, Catarina Chaves, Salvatore Cisternino, Xavier Declèves

**Affiliations:** ^1^Faculté de Pharmacie, Inserm, UMRS-1144, Optimisation Thérapeutique en Neuropsychopharmacologie, Université de Paris, Paris, France; ^2^Department of Chemical and Biological Engineering, University of Wisconsin-Madison, Madison, WI, United States; ^3^BrainPlotting, Paris, France; ^4^Service Pharmacie, Assistance Publique Hôpitaux de Paris (AP-HP), Hôpital Universitaire Necker – Enfants Malades, Paris, France; ^5^Biologie du médicament et toxicologie, Assistance Publique Hôpitaux de Paris (AP-HP), Hôpital Universitaire Cochin, Paris, France

**Keywords:** TRPV1, TRPV2, TRPV4, interspecies differences, blood–brain barrier, brain endothelial cell

## Abstract

Transient receptor potential vanilloid 1-4 (TRPV1-4) expression and functionality were investigated in brain microvessel endothelial cells (BMEC) forming the blood–brain barrier (BBB) from rat and human origins. In rat, Trpv1-4 were detected by qRT-PCR in the brain cortex, brain microvessels, and in primary cultures of brain microvessel endothelial cells [rat brain microvessel endothelial cells (rPBMEC)]. A similar *Trpv1-4* expression profile in isolated brain microvessels and rPBMEC was found with the following order: *Trpv4* > *Trpv2* > *Trpv3* > *Trpv1*. In human, TRPV1-4 were detected in the BBB cell line human cerebral microvessel endothelial cells D3 cells (hCMEC/D3) and in primary cultures of BMEC isolated from human adult and children brain resections [human brain microvascular endothelial cells (hPBMEC)], showing a similar *TRPV1-4* expression profile in both hCMEC/D3 cells and hPBMECs as follow: *TRPV2* > > *TRPV4* > *TRPV1* > *TRPV3*. Western blotting and immunofluorescence experiments confirmed that TRPV2 and TRPV4 are the most expressed TRPV isoforms in hCMEC/D3 cells with a clear staining at the plasma membrane. A fluorescent dye Fluo-4 AM ester was applied to record intracellular Ca^2+^ levels. TRPV4 functional activity was demonstrated in mediating Ca^2+^ influx under stimulation with the specific agonist GSK1016790A (ranging from 3 to 1000 nM, EC_50_ of 16.2 ± 4.5 nM), which was inhibited by the specific TRPV4 antagonist, RN1734 (30 μM). In contrast, TRPV1 was slightly activated in hCMEC/D3 cells as shown by the weak Ca^2+^ influx induced by capsaicin at a high concentration (3 μM), a highly potent and specific TRPV1 agonist. Heat-induced Ca^2+^ influx was not altered by co-treatment with a selective potent TRPV1 antagonist capsazepine (20 μM), in agreement with the low expression of *TRPV1* as assessed by qRT-PCR. Our present study reveals an interspecies difference between Rat and Human. Functional contributions of TRPV1-4 subtype expression were not identical in rat and human tissues reflective of BBB integrity. TRPV2 was predominant in the human whereas TRPV4 had a larger role in the rat. This interspecies difference from a gene expression point of view should be taken into consideration when modulators of TRPV2 or TRPV4 are investigated in rat models of brain disorders.

## Introduction

During the last decade, the transient receptor potential (TRP) channels were identified as promising pharmacological targets and some drug candidates targeting TRP have even already entered clinical trials or been approved as drugs for treating neuropathic pain, epilepsia or heart failure (Moran, 2018). Once activated possibly by heat, mechanical/shear stress, various endogenous and exogenous molecules, some TRP channels expressed at the plasma membrane increase the influx of inorganic monovalent or divalent cations (Na^+^, K^+^, Ca^2+^, Mg^2+^), triggering several downstream cell functions depending on the cell type (Rodrigues et al., 2016). Indeed, the main knowledge of TRP functions and agonists come from their expression in sensory electrically excitable cells such as neurons and their role in the nociception and pain (Mickle et al., 2015). Recently, more studies have been done and evidenced the TRP expression and their ability in modulating the functionalities of non-excitable cells such as endothelial cells using various chemical agonists and antagonists, including a requirement in vasorelaxation and vascular permeability (Kassmann et al., 2013; Grace et al., 2014; Ambrus et al., 2017). TRP channels of the vanilloid subfamily (TRPV) are divided into 6 isoforms (TRPV1-6). TRPV5 and TRPV6 are mainly expressed in intestinal and renal epithelia (Peng et al., 2018) while transient receptor potential vanilloid 1-4 (TRPV1-4) are mostly expressed in diverse cell types of the CNS, exocrine organs, skin, eye, lung, heart, and blood vessels (Steinritz et al., 2018).

Transient receptor potential vanilloid 1-4 have been evidenced in peripheral endothelial cells where they modulate the vascular tone through the release of factors that induce relaxation of smooth muscle cells (Marrelli et al., 2007), play a role in modulating endothelial permeability (Villalta and Townsley, 2013), participate in angiogenesis of peripheral vascular (Smani et al., 2018), and sense hemodynamic and chemical changes (Kohler et al., 2006). Ca^2+^ modulation has been known to be an important factor in maintaining the function of brain microvessel endothelial cells (BMEC) forming blood–brain barrier (BBB) (Brown and Davis, 2002; De Bock et al., 2013). By modulating Ca^2+^ trafficking into BMEC, a growing evidence has been achieved that TRP channels may have a critical role on the BBB properties including its permeability, angiogenesis and inflammatory responses (Berrout et al., 2012; Huang et al., 2020). However, the expression or functionality of TRPV in BBB was much less studied than in non-cerebral endothelial cells. The expression or functionality of TRPV in BMEC forming the BBB was much less studied than in non-cerebral endothelial cells although there is growing evidence that TRP channels may have a critical role at the BBB by modulating Ca^2+^ trafficking into BMEC. Dysfunctions of the BBB are increasingly recognized as a cause or a consequence in the pathophysiology of diverse brain pathologies such as stroke, neurodegenerative and psychiatric diseases (Sweeney et al., 2019; Yang et al., 2019; Kealy et al., 2020; Yu et al., 2020). A dysfunctional BBB can thus be targeted with new drug candidates, which would be a promising way for treating brain pathologies. However, pre-clinical proof of concept studies of such drug candidates are usually developed with rodent models, thus raising the question of interspecies differences regarding the BBB equipment between rodents and human. Moreover, TRPV isoforms can be specifically activated by various physical and/or chemical stimuli such as heat, osmolality, and mechanical stress (Montell, 2001). Thus, the comparative expression levels of TRPV1-4 in the BBB of rodent pharmacological models and human are meaningful in clarifying the possible involvement of each isoform in physiological and pathological BBB. A whole transcriptomic analysis revealed a relative low Trpv2 expression in murine primary BMEC (Zhang et al., 2014) and rat Trpv2 was not immunolocalized in the brain vasculature (Nedungadi et al., 2012), whereas TRPV2 was shown highly expressed in the human cerebral microvessel endothelial cells D3 cells (hCMEC/D3) human cerebral microvessel endothelial cell line (Luo et al., 2019), suggesting interspecies differences in the expression of TRPV between rat and human BMEC.

The present work aims at determining TRPV1-4 expression in primary cultured rat BMEC, rat cortex and isolated cortex microvessels, as well as in primary cultured human BMEC isolated from patient biopsies and the hCMEC/D3 human cerebral microvessel endothelial cell line. Functional activity of human TRPV1-4 channels was subsequently studied in hCMEC/D3 cells.

## Materials and Methods

### Reagents and Chemicals

Cannabidiol (CBD) (1 mg/mL in methanol), tranilast (TNL), capsaicin (CAP), capsazepine (CPZ), and GSK1016790A (GSK) were all analytical grade and purchased from Sigma-Aldrich (Saint-Quentin-Fallavier, France). CaCl_2_, MgSO_4_, NaCl, NaH_2_PO_4_, NaHCO_3_, KH_2_PO_4_, and KCl were purchased from Merck (Fontenay-sous-Bois, France).

RNA extraction kits were bought from Qiagen (Courtaboeuf, France). Primers, RT-PCR reagents and Lipofectamine^®^ RNAi MAX transfection reagent were obtained from Invitrogen Life Technologies (Cergy-Pontoise, France). The Power SYBR Green PCR Master Mix was purchased from Applied Biosystems (Applied Biosystems^TM^, France). All other reagents and chemicals were purchased from Sigma.

Dulbecco’s modified Eagle’s medium (DMEM), DMEM/F12 + Glutamax, Hank’s buffered salt solution (HBSS), HEPES and Penicillin/Streptomycine were from Life Technologies (Cergy-Pontoise, France). fetal bovine serum (FBS) was from GE Healthcare Life Science. Liberase DL, bovine serum albumin (BSA) (cat. # A7906), Dextran (cat. # 31390), DNase I (cat. # DN25), Puromycine and b-FGF were from Sigma-Aldrich. plasma derived bovine serum (PDBS) was from First Link (Wolverhampton, United Kingdom).

### Isolation of Rat Brain Microvessels

The isolation of rat brain cortex microvessels was done according to a protocol reported previously (Chaves et al., 2020). CO_2_ anesthetized animals were decapitated, rat brains were removed and immediately soaked in ice-cold HBSS containing 10 mmol/L HEPES. Brain cortices were isolated, minced and homogenized, and subsequent brain homogenates were centrifuged (2000 × *g*, 10 min), the pellets were re-suspended in 17.5% 64–76 kDa dextran (Sigma Aldrich) and centrifuged one more time (4400 × *g*, 15 min). The resulting pellets were suspended in HBSS containing 1% BSA and were firstly filtered through a 100 μm nylon mesh, and then through a 20 μm nylon mesh. The retained cerebral cortex microvessels were immediately collected and stored at −80°C before analysis (diameter size around 4–6 μm).

### Cell Culture Conditions

#### Human Cerebral Microvessel Endothelial Cells D3 Cells (hCMEC/D3)

The hCMEC/D3 cell line kindly given by Cochin Institute (Paris, France). were used from passages 27 to 33 and cultured with the EndoGRO complete medium (Merck), supplemented with 5% FBS,1% streptomycin-penicillin (Gibco, Carlsbad, CA, United States), and 1 ng/mL b-FGF (Sigma) under 5% CO_2_ and 37°C as previously described (Luo et al., 2019). Culture flasks and plates were pre-coated with 150 μg/mL rat tail collagen type I. Assessment of intracellular calcium kinetics was carried out on confluent cultures in 24-well plates seeded at 5 × 10^4^ cells/cm^2^.

#### HEK-293 Cells

Human embryonic kidney HEK293 cells were cultured with DMEM (Gibco) containing 10% FBS (Thermo) and 1% streptomycin-penicillin (Gibco) under 5% CO_2_ and 37°C.

#### Primary Cultures of Rat Brain Microvessel Endothelial Cells (rPBMEC)

Adult male Sprague-Dawley rats weighing 300–350 g were purchased from Charles River laboratory (L’arbresle, France). They were housed in groups of four per cage under standard 12:12-h light/dark conditions (light from 08:00 to 20:00 h) in a temperature- and humidity-controlled room. They had access to food and water *ad libitum*. Rats were acclimated for 7 days before experimentation. The care and treatment of animals conformed to the standards and guidelines promulgated by the European Communities Council Directive (86/609/EEC). The protocol was approved by the ethics review committee of Paris Descartes University (approval n°12-185/12-2012). Rats were deeply anesthetized by intraperitoneal administration of diazepam (5 mg/mL) and ketamine (100 mg/mL). They were perfused transcardially with Buffer 1 (HBSS, 10 mmol/L HEPES) at room temperature (RT) for 3 min. All subsequent steps were done on ice except when indicated. The cortex was dissected, cleared from adhering white matter and meninges and gently crushed in a Petri dish using a glass slide. Tissue pieces from two cortices were pooled, rinsed in Buffer 1, then centrifuged (2 min, 600 g) and resuspended in 10 mL of DMEM containing 10 mM HEPES, 0.4 WU/mL Liberase DL and 100 U/mL DNase I. Digestion was performed for 40 min at 37°C with gentle mechanical trituration with a 10 mL pipette (at 10 min), then with a P1000 pipet tip (at 20 min) and finally with a roded glass Pasteur pipette (at 30, 35, and 40 min), to obtain an homogenate with a creamy texture and almost no visible remaining piece. Digestion was stopped by adding 30 mL of DMEM + 10% FBS and the homogenate was centrifuged (5 min, 1,000 × *g*). The supernatant was discarded and the pellet was resuspended in 25 mL of Buffer 1 + 18% BSA and centrifuged (15 min, 2,000 × *g*). The compact myelin disk was eliminated and the pellet resuspended in 50 mL of Buffer 1 + 1% BSA. This suspension was filtered on a 10 μm nylon mesh (cat. # NY1004700, Millipore). Cells from the filtrate were pelleted, resuspended in DMEM + 10% FBS and counted. Cells were then seeded in 12-wells plate (1.5 × 10^6^/well) in complete medium (DMEM/F12 + Glutamax, 20% PDBS, 10 U/mL penicillin/streptomycin, 80 μg/mL heparin, 5 ng/mL b-FGF) containing 4 μg/mL puromycin. After 3 days, medium was changed for complete medium without puromycin and cells were grown until confluency.

#### Primary Cultures of Human Brain Microvascular Endothelial Cells (hPBMEC)

All human samples were provided by BrainPlotting (iPEPS, Institut du Cerveau et de la Moelle épinière, Hôpital Universitaire de la Pitié-Salpêtrière, Paris, France) in partnership with Sainte-Anne Hospital, Paris (neurosurgeon Dr. Johan Pallud) and harvested during tumor scheduled resection surgery with written informed consent from the patients (authorization number CODECOH DC-2014-2229). Human brain microvessels were obtained from surgical resections of three patients: a 70-years-old female suffering from Glioblastoma, a 2-year-old child suffering from glioma and a 8-year-old child suffering from astrocytoma. Microvessels were isolated from resections of tumor, peritumoral or healthy brain tissue using an enzymatic procedure (Chaves et al., 2020) adapting methods previously published for rats (Perriere et al., 2005, 2007). Briefly, tissue samples were carefully cleaned from meninges and excess of blood; then, an enzymatic mix was used to dissociate the tissues and microvessels were isolated by retention on a 10 μM mesh. Cells were cultured in EBM-2 medium (Lonza, Basel, Switzerland) supplemented with 20% serum and growth factors (Sigma) (Perriere et al., 2005, 2007). After seeding brain capillaries in petri dishes, brain primary microvascular endothelial cells were shortly amplified and seeded (P1) on Transwell (Corning) with microporous membranes (pore size: 0.4 μm) in monoculture (CC205, CC206, and CC216) or in co-culture with the same patient’s fresh primary human cultured astrocytes (CC216). Dry cell pellets were stored at −80°C before RT-qPCR experiments.

### RNA Isolation, Reverse Transcription and Quantitative Real Time PCR (qPCR)

Total RNA was purified using the RNeasy Mini kit (Qiagen GmbH, Hilden, Germany). The concentration and purity of the RNA samples were determined using the NanoDrop^®^ ND-1000 instrument (NanoDrop Technologies, United States). Reverse transcription of total RNA was achieved in a thermocycler (PTC-100 programmable thermal controller, MJ research INC, United States) using the following conditions: 25°C for 10 min, then at 42°C for 30 min and at 99°C for 5 min, as previously reported (Dauchy et al., 2009). cDNAs were stored at −80°C. Gene expression was determined by SYBR Green fluorescence detection using an ABI Prism 7900 HT Sequence Detection System (Applied Biosystems, Foster City, CA, United States) as previously reported (Luo et al., 2019). Samples were run in duplicate. Primers (Table 1) were designed using OLIGO 6.42 software (MedProbe, Norway). The lower the cycle threshold (Ct) value the higher the amount of mRNA, RT negative controls and no-template controls had Ct values > 40. RT negative controls and no-template controls had Ct values > 40. cDNAs from HEK293 cells were used to validate primers of human genes of interest. The relative expression of each gene of interest X as compared to the expression of the housekeeping gene encoding TATA box-binding protein (*TBP*) was calculated as 2^–(Ct(X)–Ct(TBP))^. Gene expression of genes having a Ct higher than 33 was not quantified.

**TABLE 1:** Primers list for TRPVs channels and TBP applied in human and rat.

Gene	Forward primer (5′-3′)	Reverse primer (3′-5′)	Length (bp)
*TBP or Tbp*	TGCACAGGAGCCAAGAGTGAA	CACATCACAGCTCCCCACCA	132
*Human TRPV1*	ACTGCCATCATCACTGTCATCT	CTTCACAGCCAACAGGTCTACCA	83
*Human TRPV2*	CGTGCAGATCCCCTTCGAGA	TGGGCAACTTGTGATACAGATGG	85
*Human TRPV3*	TACGTGGCTGAGGGAGAG	ATCGTGGCGTGAAGTCCA	92
*Human TRPV4*	TCAGGGAATCACAGTTGGC	AGCTCTTCATTGATGGATTCTT	82
*Rat Trpv1*	CTCGCTCTCCGCCATTCTTGC	TGTGCGTGACTTATGGGAGATGT	88
*Rat Trpv2*	ACCACCAAAAAGTGTCCCTTCTC	CGGCCTCTTTAGGAGTCTCACC	94
*Rat Trpv3*	GCTCCAGCCTGTGTTGAAAAT	CCTCCCAGTCCCTTGCTAAAG	90
*Rat Trpv4*	CCCCCTCAGCTCTTCACGG	CCCCACCCTGTTCCACTTTTTC	114

### Western Blot

Human cerebral microvessel endothelial cells D3 cells were washed twice with cold phosphate-buffered saline (PBS) on ice, then the protein lysis buffer (150 mM NaCl, 50 mM Tris–HCl pH 7.4, 0.5% Triton X100, 0.5% sodium deoxycholate), and protease inhibitor cocktail (complete^®^, Sigma) was added. The Bradford assay was applied to quantify the protein concentration (BSA as a standard). Total proteins were separated on a 7.5% SDS-polyacrylamide gel and transferred to polyvinylidene difluoride (PVDF) membranes (Bio-Rad), blocked for 2 h with 5% non-fat dry milk. Membranes were incubated overnight with a mouse anti-human TRPV2 primary antibody (1/250, sc-390439, Santa Cruz Biotechnology, Dallas, TX, United States), a polyclonal rabbit anti-human TRPV4 primary antibody (1/200, ab94868, Abcam) or a monoclonal mouse anti-human β-actin primary antibody (1/3000, Millipore). A secondary anti-mouse or anti-rabbit IgG conjugated to HRP (1/2000, Santa Cruz Biotechnology) was then incubated for detection using an ECL plus Western Blot Detection System (GE Healthcare, Velizy, France).

### Confocal Imaging

Human cerebral microvessel endothelial cells D3 cells were cultured on a 8-well ibidi μ-Slide (1.5 polymer coverslip, tissue culture treated from CliniSciences, Nanterre, France). At 80% confluence, cells were fixed with PBS containing 3.2% paraformaldehyde and permeabilized with 0.2% Triton-X-100 (Sigma) in PBS for 10 min. After 30 min in blocking solution (0.2% Triton-X-100, 1% BSA and 10% goat serum containing PBS) at RT, cells were incubated with the rabbit anti-human TRPV2 primary antibody (1:250, Thermo Fisher Scientific, Ref: PA1-18346), rabbit anti-human TRPV4 primary antibody (1:100, ab94868, Abcam), rabbit anti-human Claudin-5 primary antibody (1:250, Santa Cruz Biotechnology) or rabbit anti human VE-Cadherin primary antibody (1:500, Enzo Life Sciences) overnight at 4°C. After washing with PBS, μ-slides were incubated for 2 h at RT with a goat anti-rabbit-555 (1:500, Santa Cruz Biotechnology) and nuclei were stained with Hoechst 33342 (1:10000, Thermo Fisher Scientific, France). Negative control cells were incubated omitting primary antibodies. Visualization of the proteins was performed using a Zeiss LSM 510 Meta confocal microscope (Oberkochen, Germany).

### Intracellular Ca^2+^ Signal Measurements

Fluorescence measurement of intracellular Ca^2+^ concentration was performed as described previously (Luo et al., 2019). hCMEC/D3 cells grown to confluency in 24-well plate were loaded with 2 μM of Fluo-4-AM (λ_*ex*_ = 496 nm, λ_*em*_ = 516 nm, F14201, Thermo Fisher Scientific) for 45 min at 37°C in Hank’s buffer (5.37 mM KCl, 0.44 mM KH_2_PO_4_, 4.2 mM NaHCO_3_, 137.9 mM NaCl, 0.34 mM Na_2_HPO_4_, 1.5 mM CaCl_2_, 0.9 mM MgSO_4_, 5.56 mM D-Glucose at pH 7.4, all from Sigma, 500 μL/well). Cells were then washed and replaced with HBSS (500 μL/well). After 10 min of incubation at 37°C, fluorescence in the 24-well plates were read using a Victor^®^ X2 fluorescent microplate reader (PerkinElmer, Villebon, France).

Fluorescence was determined with diverse activators of TRP channels. Cells were also pre-treated with TRP antagonists (capsazepine 10 and 20 μM, tranilast 50 μM, RN1734 30 μM) for 5 min before adding TRP agonists (GSK1016790A ranging from 3 to 1000 nM, capsaicin 1 and 3 μM, cannabidiol 15 μM) and the fluorescent signals were then recorded in the presence of fixed concentration of TRP antagonists. All TRP activators and antagonists were dissolved with 100% DMSO or ethanol as stock solutions and the final solvent concentration in treated cells did not exceed 1%, a solvent concentration that had no effect on Ca^2+^ entry as compared to control cells without any solvent (data not shown). Data were normalized to the control group, which is expressed as F1/F0, where F0 is the average fluorescence of the control group (no activator of TRP) and F1 is the actual fluorescence at the corresponding time for the control group (in this case the average ratio for the control group is 1 or 100%) or the treated group.

To explore heating-induced Ca^2+^ influx, hCMEC/D3 cells were plated in 24-wells plate and loaded with Fluo-4-AM. The plate was then placed in the detecting room of Victor^TM^ X2 fluorescent microplate reader with automatically heating system. The plate was heating from RT to 50°C. The temperature and fluorescence were recorded at the same time by the Victor^TM^ X2 in the presence or not of the specific TRPV antagonist. Data was expressed as ratio normalized to the average of fluorescence intensity at RT in each well.

### Statistical Analysis

Data are expressed as mean value ± SEM (standard error of the mean). Statistical analysis was performed using ANOVA with Dunnett *a posteriori* test to compare different groups with the control. An unpaired *t*-test was applied between two groups. *p* value < 0.05 was considered statistically significant. To calculate the EC_50_ of the agonist (GSK1016790A), the concentration-response data were fitted to a logistic function as follows: *Y* = Bottom + (Top−Bottom)/(1 + 10^(logEC50–X)^); where X is the log of the agonist concentration. Data fitting was performed in GraphPad Prism 5.01.

## Results

### Thermo-Sensitive *Trpv1-4* Gene Expression in Rat Cortex, Brain Microvessels and rPBMEC

**FIGURE 1:**
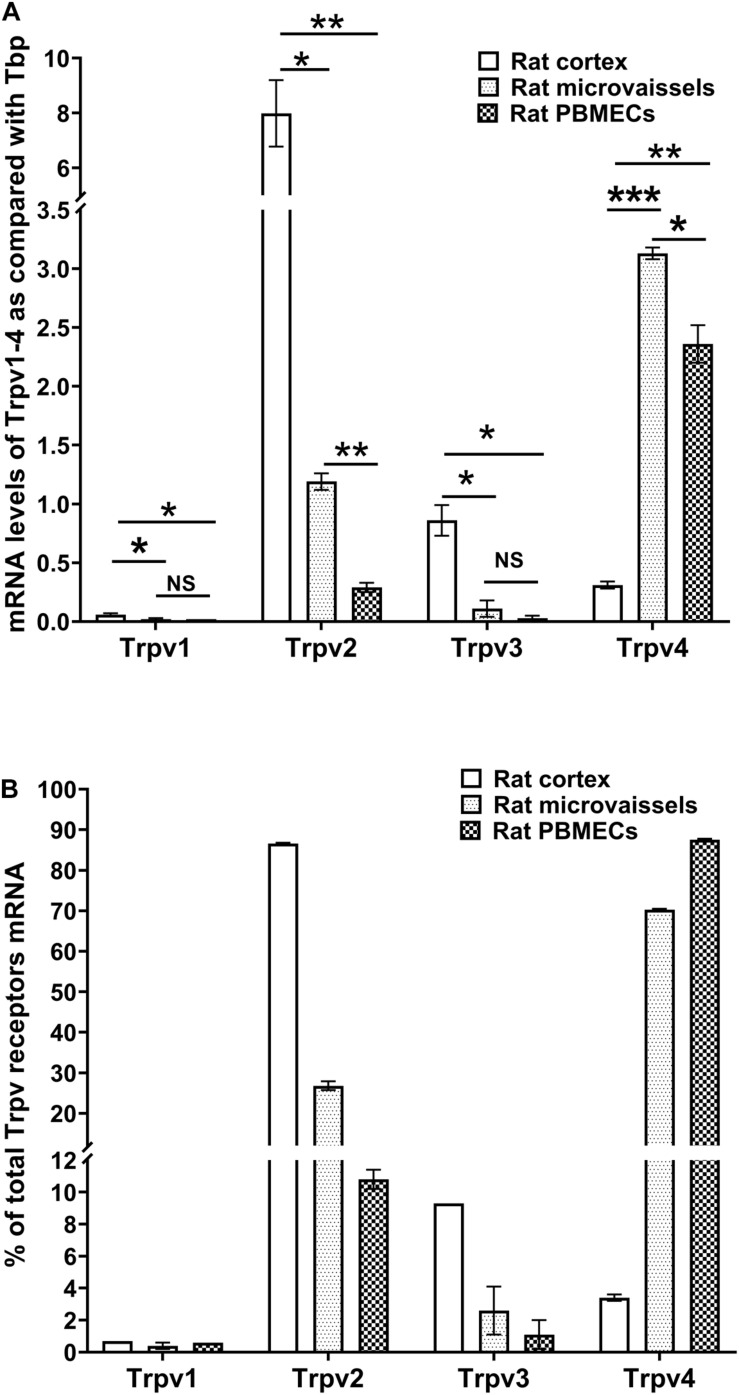
Expression of thermo-sensitive Trpv in rat cortex, rat microvessels and BBB endothelial cells. **(A,B)** mRNA levels of *Trp1-4* were detected by qRT-PCR in rat brain cortex, rat brain isolated microvessels and primary cultures of rat brain microvessel endothelial cells (see section “Materials and Methods”). Data are expressed as ratio (mean ± SEM) of *Trpv* mRNA levels compared with those of the endogenous housekeeping gene *Tbp* in each sample from three different RNA extractions **(A)** or expressed as a percentage of total *Trpv1-4* expression set at 100% **(B)**. Inter-group comparisons were performed by ANOVA with Dunnett *a posteriori* test, NS not significant, *p < 0.05, **p < 0.01, ***p < 0.001.

We measured the relative expression of *Trpv1-4* by qRT-PCR in primary cultured rat brain microvessel endothelial cells (rPBMEC) (Figure 1). *Trpv1* mRNA levels were barely detectable in whole cortex, brain microvessels and rPBMEC (Figure 1A). *Trpv2* expression was much higher in the whole cortex than in both brain microvessels and rPBMEC (7.99 ± 1.21, 1.19 ± 0.07, and 0.29 ± 0.04, respectively; *p* < 0.05 rat cortex vs. microvessels, *p* < 0.01 rat cortex vs. rPBMEC) and similar results were obtained for *Trpv3* (0.86 ± 0.13, 0.11 ± 0.07, 0.03 ± 0.02, respectively; *p* < 0.05 rat cortex vs. microvessels, *p* < 0.05 rat cortex vs. rPBMEC). Among *Trpv1-4*, *Trpv4* was the most highly expressed gene in brain microvessels and rPBMEC (3.13 ± 0.05 and 2.36 ± 0.16, respectively; *p* < 0.001 rat cortex vs. microvessels, *p* < 0.01 rat cortex vs. rPBMEC) while its expression in rat cortex was far below that of *Trpv2*. Meanwhile, no significant difference was observed for the gene expression of *Trpv1* and *Trpv3* between brain microvessels and rPBMEC (Figure 1A), while a significant decrease of *Trpv2* and Trpv 4 gene expression was observed in rPBMEC compared with that in brain microvessels (Figure 1A, *p* < 0.01, *p* < 0.05 for *Trpv2* and *Trpv4*, respectively, rat microvessels vs. rPBMEC). The *Trpv1-4* gene expression profile expressed as a percentage of total *Trpv* genes expressed was similar in brain microvessels and rPBMEC with the following rank order: *Trpv4* > *Trpv2* > *Trpv3* > > *Trpv1* (Figure 1B). This is dramatically different from the *Trpv1-4* gene expression profile in the whole brain cortex with the following rank order: *Trpv2* > *Trpv3* > *Trpv4* > > *Trpv1*.

### Thermo-Sensitive *TRPV1-4* Gene Expression in Human BMEC

**FIGURE 2:**
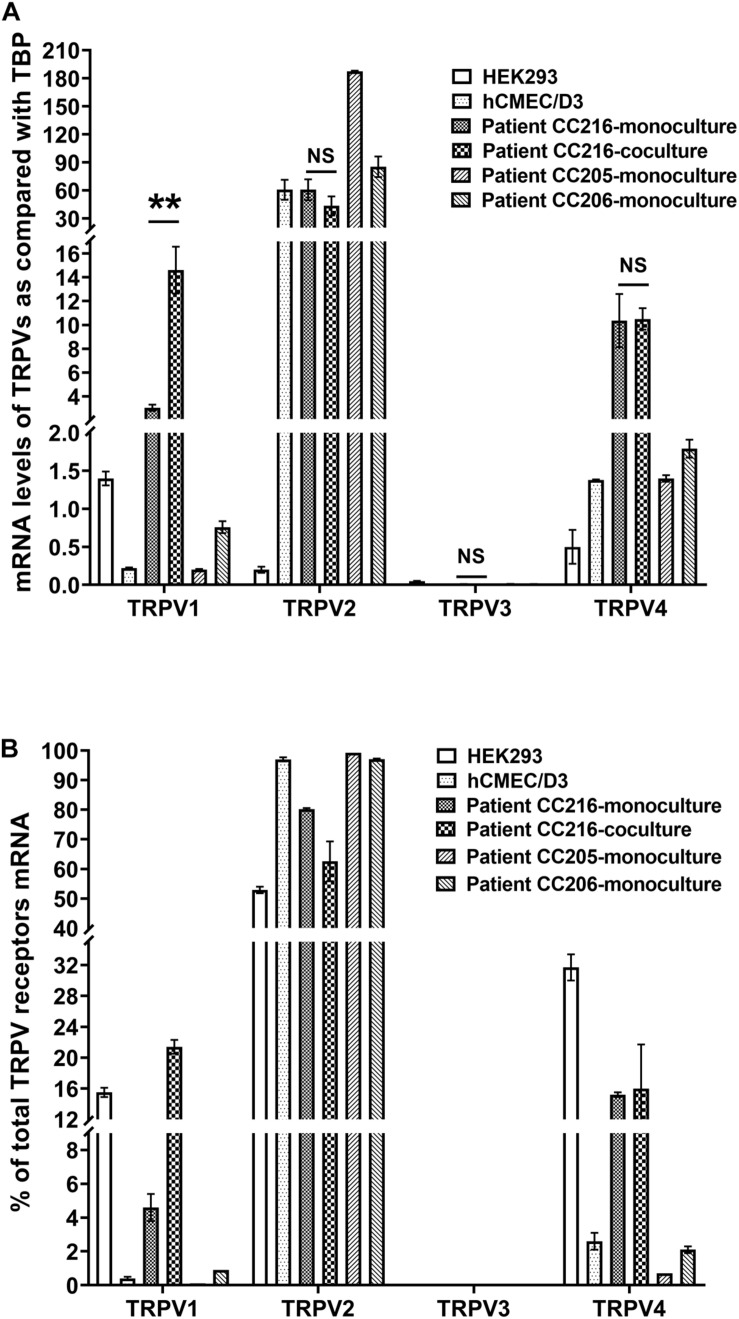
Expression of thermo-sensitive Trpv in human BMEC. **(A,B)** mRNA levels of *TRPV1-4* were detected by qRT-PCR in primary cultures of brain microvessel endothelial cells obtained from patients (see section “Materials and Methods”) and in hCMEC/D3 cells. Data are expressed as ratio (mean ± SEM) of TRPV1-4 mRNA levels compared with those of the endogenous housekeeping gene *TBP* in each sample from three different RNA extractions **(A)** or expressed as a percentage of total *TRPV1-4* expression set at 100% **(B)**. When comparing TRPV1-4 expression in human BMEC culturing alone or together with astrocytes isolated from the same patient, statistical significance was determined by an unpaired *t*-test, NS, not significant, **p < 0.01.

We measured the relative expression of *TRPV1-4* by qRT-PCR in hCMEC/D3 cells and primary cultured human brain microvascular endothelial cells (hPBMEC) from three different brain tumor biopsies (Figure 2A). *TRPV1* mRNA levels were easily detected in hPBMEC of the adult patient culture CC216 and, albeit at a lower level, in hCMEC/D3 and hPBMEC from the two children cultures CC205 and CC206 (3.05 ± 0.26, 0.22 ± 0.01, 0.21 ± 0.01, and 0.76 ± 0.08, respectively, Figure 2A). It is noteworthy that *TRPV1* expression was increased significantly by 4.8-fold (from 3.05 ± 0.26 to 14.6 ± 2.0, *p* < 0.01) when cells were co-cultured with isolated primary astrocytes from the same patient, while no effect was found for the other 3 TRPV isoforms (Figure 2A). *TRPV2* was by far the most highly expressed gene in hCMEC/D3 and hPBMEC cells [*TRPV2* data for CC205 and CC206 was also shown in Luo et al. (2019)]. *TRPV4* was expressed at moderate levels in hPBMEC from the three patients (10.36 ± 2.23, 1.35 ± 0.04, and 1.79 ± 0.12) and hCMEC/D3 (1.38 ± 0.01) while *TRPV3* mRNA were barely detectable in all cells. A similar *TRPV1-4* gene expression profile expressed as a percentage of total *TRP* gene expressed was observed in hCMEC/D3 cells and hPBMEC with the following rank order: *TRPV2* > > *TRPV4* > *TRPV1* > > *TRPV3* (Figure 2B).

### Protein Expression of TRPV2 and TRPV4 in hCMEC/D3

**FIGURE 3:**
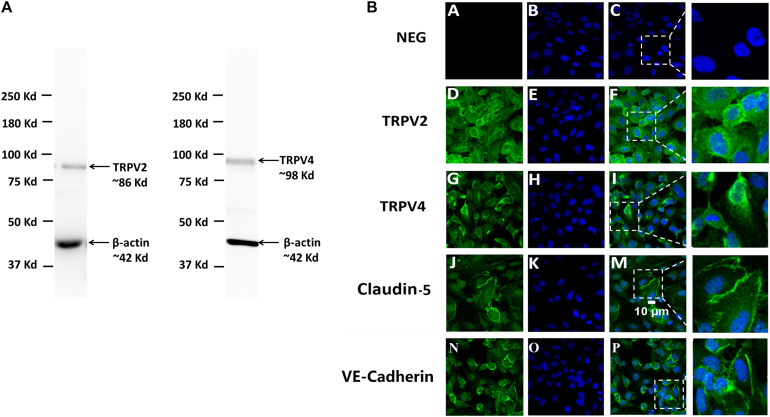
Protein expression of TRPV2 and TRPV4 in hCMEC/D3. **(A)** Expression of TRPV2 and TRPV4 determined by Western blot of total crude proteins obtained from two different protein extractions of hCMEC/D3. β-actin served as a loading control housekeeping protein. **(B)** Confocal immunofluorescence localization of TRPV2 and TRPV4 in hCMEC/D3. TRPV2 or TRPV4 (green) and nuclei (blue) were stained in hCMEC/D3 as described in section “Materials and Methods” (second D, E, F and third G, H, I row panels). Claudin-5 (green) and nuclei (blue) were stained in hCMEC/D3 (fourth row panel J, K, M). VE-Cadherin (green) and nuclei (blue) were stained in hCMEC/D3 (fifth row panel N, O, P). Control cells (NEG) were stained omitting the primary antibodies (first row panel A, B, C).

The expression of TRPV2 and TRPV4, the two genes with the highest expression at the mRNA level in human brain endothelial cells, was then studied at the protein level. Western blot analysis showed that TRPV2 and TRPV4 proteins were expressed in hCMEC/D3 cells where they are detected in a single band at a molecular weight around 80–90 kDa, in agreement with the predicted value of TRPV2 and TRPV4 molecular weights (Figure 3A). This was confirmed by immunostaining and confocal microscopy which revealed a specific expression of both TRPV2 and TRPV4 at the plasma membrane of hCMEC/D3 cells as well as in intracellular compartments concentrated in the perinuclear region (Figure 3B). Omitting the primary antibody resulted in the absence of any detectable fluorescence signal (NEG in Figure 3B). The tight junction protein Claudine-5 was used as a positive control for brain endothelial cell phenotype as it was reported to be expressed in hCMEC/D3 cells (Weksler et al., 2005).

### Functional Activity of TRPV in hCMEC/D3

Transient receptor potential vanilloid 1, TRPV2, and TRPV4 functional activity was then investigated in hCMEC/D3 cells. Ionomycin, which triggers an immediate increase of the intracellular Ca^2+^ concentration, was used as a positive control (2 μM ionomycin) in all Ca^2+^ influx experiments.

**FIGURE 4:**
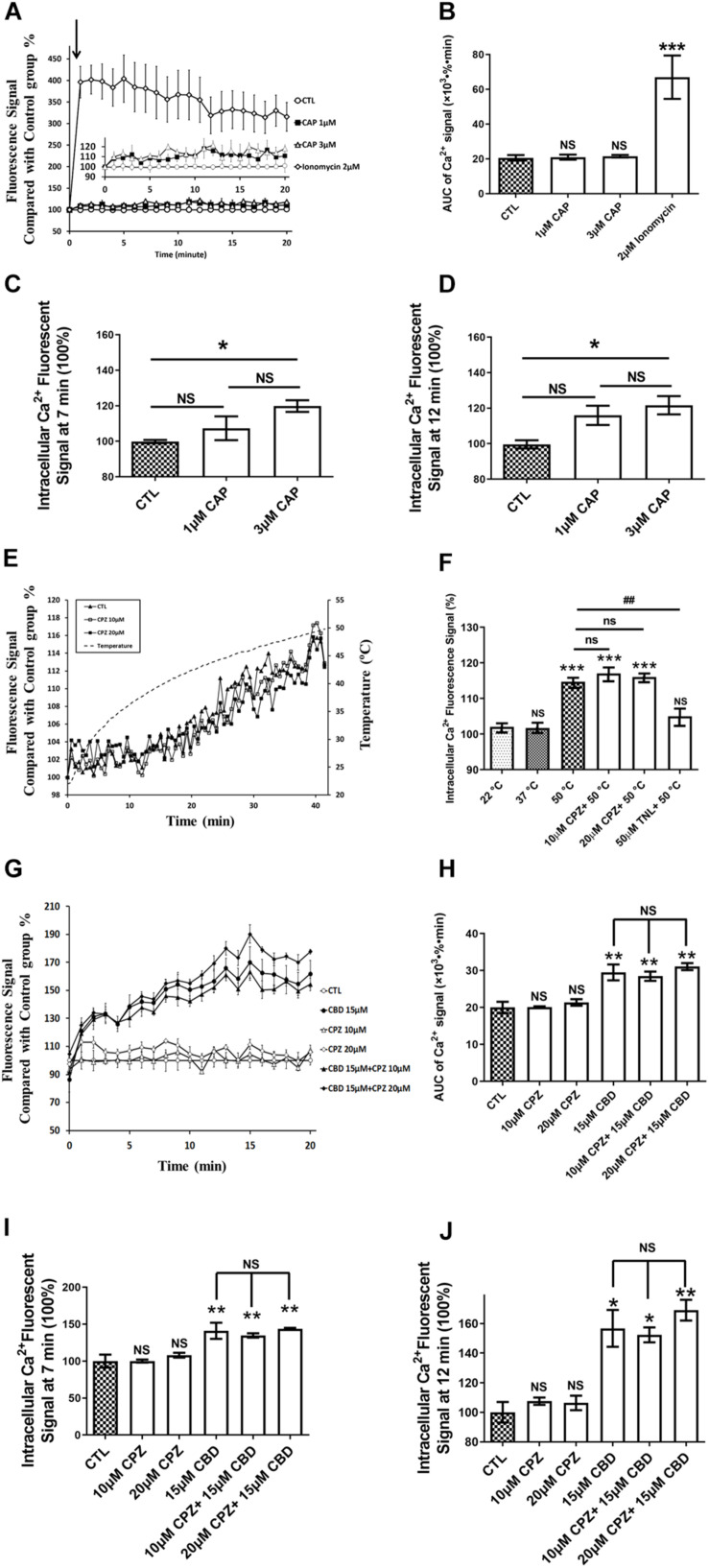
TRPV1 is not functional in hCMEC/D3. **(A)** Representative time course of [Ca^2+^]_*i*_ in hCMEC/D3 under stimulation of TRPV1 by capsaicin (CAP). Cells were plated in 24-wells plate and loaded with Fluo-4-AM as described in section “Materials and Methods.” CAP or ionomycin (as positive control of Ca^2+^ entry) was added and the fluorescence was recorded. **(B)** Change of the area under curve (AUC) over the 20 min obtained from the experiment shown in panel **(A)**. **(C)** Change in [Ca^2+^]_*i*_ at *T* = 7 min obtained from the experiment shown in panel **(A)**. **(D)** Change in [Ca^2+^]_*i*_ at *T* = 12 min obtained from the experiment shown in panel **(A)**. **(E)** Change of [Ca^2+^]_*i*_ upon heat exposure of hCMEC/D3 for 40 min from room temperature to 50°C. Cells were plated in 24-wells plate and loaded with Fluo-4-AM. The fluorescence was recorded in the presence or not of the specific TRPV1 antagonist capsazepine (CPZ; 10 or 20 μM). **(F)** Changes in [Ca^2+^]_*i*_ in hCMEC/D3 at 22, 37, or 50°C with or without CPZ or Tranilast (TNL, 50 μM) derived from panel **(E)**. **(G)** Representative time course of [Ca^2+^]_*i*_ in hCMEC/D3 stimulated by the TRPV1-2 agonist cannabidiol (CBD, 15 μM) and pre-treated or not with the TRPV1 specific antagonist CPZ. Cells were loaded with Fluo-4-AM. CBD was added and the fluorescence was recorded. When applying CPZ, cells were pre-treated with 10 or 20 μM CPZ for 5 min before incubation with 15 μM CBD and the fluorescence was recorded in the persistent presence of 10 or 20 μM CPZ. **(H)** Change of the area under curve (AUC) over the 20 min obtained from the experiment shown in panel **(G)**. **(I)** Change in [Ca^2+^]_*i*_ at *T* = 7 min obtained from the experiment shown in panel **(G)**. **(J)** Change in [Ca^2+^]_*i*_ at *T* = 12 min obtained from the experiment shown in panel **(G)**. Data are expressed as mean ± SEM and inter-group comparisons were performed by ANOVA with Dunnett *a posteriori* test. *p < 0.05, **p < 0.01, ***p < 0.001 compared with CTL or 22°C groups or among indicated group.

The AUC of intracellular Ca^2+^ levels during 20 min were not significantly affected when cells were exposed for 20 min to 1 or 3 μM CAP, a highly specific and efficient agonist of TRPV1 (Figures 4A,B). By contrast, CAP, at lower concentrations than those used in our study, were high enough to fully activate human TRPV1 (Binshtok et al., 2007). In addition to compare the AUC of intracellular Ca^2+^ levels during 20 min, we further compared the intracellular Ca^2+^ levels at specific time (Figures 4C,D). Interestingly, a significant statistical difference between the control group and 3 μM CAP group at 7 min (*p* = 0.019), 11 min (*p* = 0.041), 12 min (*p* = 0.049), and 16 min (*p* = 0.045) were found (Figures 4C,D). However, no significant difference of Ca^2+^ influx at any specific times was found when cells exposed to 1 μM CAP (Figures 4C,D). Ca^2+^ flux levels were not significantly altered when cells exposed to CAP from 1 to 3 μM (Figures 4C,D). Meanwhile, a significant 3.3-fold increase of Ca^2+^ influx was observed in the positive control group treated with 2 μM ionomycin (Figures 4A,B, *p* < 0.001 compared with the control group). TRPV1 is a known thermo-sensitive channel (Mergler et al., 2010). Therefore, we asked whether CPZ, a well-known TRPV1 antagonist, was able to block the heat-induced increase of intracellular Ca^2+^ concentration. As shown in Figures 4E,F, exposing cells continuously from RT to 50°C for 40 min resulted in a significant increase in intracellular Ca^2+^ but this Ca^2+^ flux was not altered by co-treatment with CPZ (10 or 20 μM; Figure 4E), in agreement with the low expression of *TRPV1* assessed by qRT-PCR and the weak TRPV1 activation by CAP. Conversely, heat-induced Ca^2+^ influx could be blocked by TNL, a selective TRPV2 antagonist. Exposing the cells to CBD, a highly potent activator of TRPV2 and to a lesser extent of TRPV1, resulted in an increased Ca^2+^ influx in hCMEC/D3, an effect which was not reversed by CPZ (Figures 4G,H). Similar trends were demonstrated when comparing the Ca^2+^ influx at specific time (Figures 4I,J).

**FIGURE 5:**
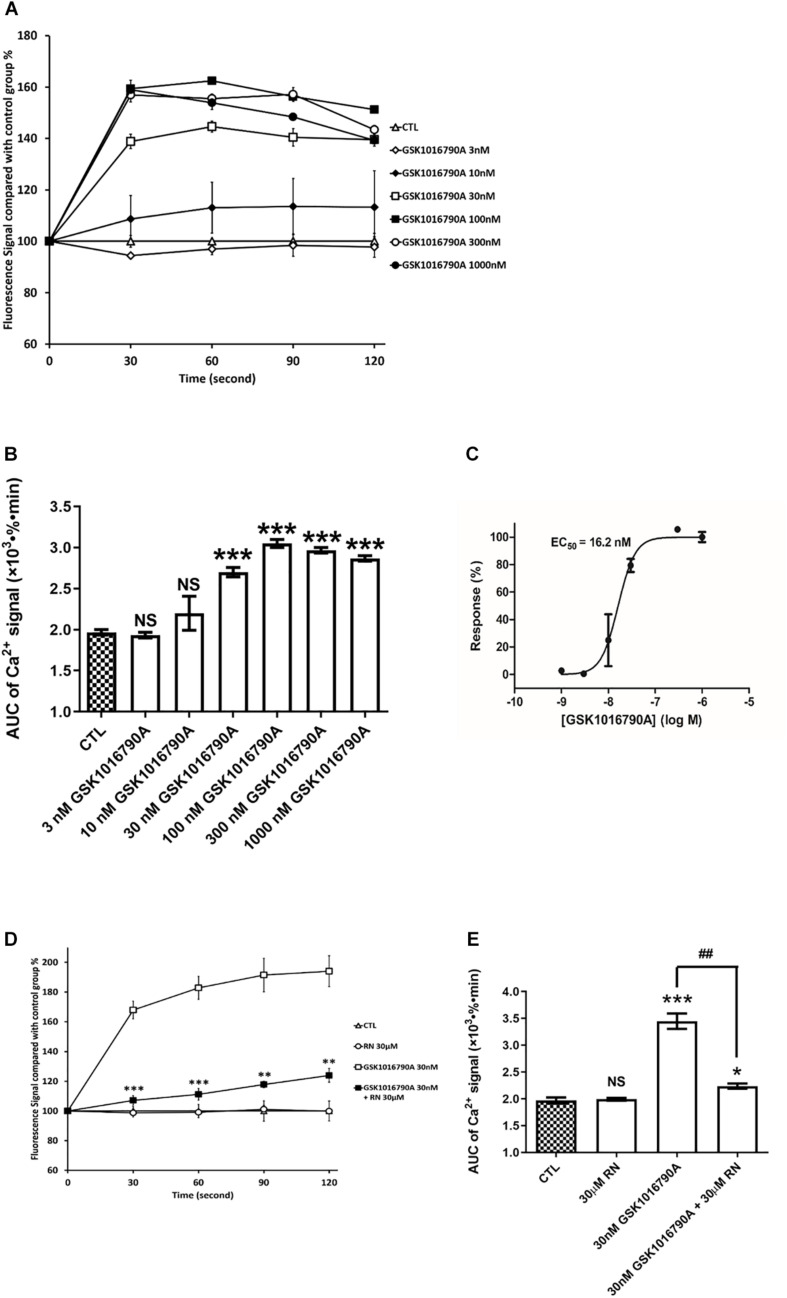
TRPV4 is functional in hCMEC/D3. **(A)** Representative time course of [Ca^2+^]_*i*_ in hCMEC/D3 stimulated by the TRPV4 agonist GSK1016790A. Cells were plated in 24-wells plate and loaded with Fluo-4-AM as described. GSK1016790A was added and the fluorescence was recorded. **(B)** Change of the area under curve (AUC) over the 120 s obtained from the experiment shown in panel **(A)**. **(C)** Concentration-response relationship of GSK1016790A. Measurements were obtained from panel **(B)**. **(D)** Representative time course of [Ca^2+^]_*i*_ in hCMEC/D3 stimulated by GSK1016790A and pre-treated or not with the TRPV4 specific antagonist RN1734 (RN). When applying RN, cells were pre-treated with 30 μM RN for 5 min and the fluorescence was recorded in the persistent presence of 30 μM GSK1016790A. **(D)** Change of the area under curve (AUC) over the 120 s obtained from the experiment shown in panel **(C)**. Data are expressed as mean ± SEM. For panels **(B,E)**, inter-group comparisons were performed by ANOVA with Dunnett *a posteriori* test, NS, not significant, *p < 0.05, ***p < 0.001 compared with CTL group, ##*p* < 0.01 compared with 30 nMGSK1016790A group, *n* = 3 in triplicate. For panel **(D)**, inter-group comparisons were performed by ANOVA with Dunnett *a posteriori* test, *p < 0.01, **p < 0.01, ***p < 0.001 compared with GSK1016790A group.

To assess TRPV4 activity in hCMEC/D3, we applied GSK1016790A (GSK), a highly selective and potent agonist of TRPV4. GSK induced a dramatic and rapid concentration-dependent increase in Ca^2+^ levels (Figure 5A). A maximal increase in intracellular Ca^2+^ levels (1.6-fold) was obtained with GSK ≥100 nM (Figure 5B). An apparent concentration-effect relationship was observed with an EC_50_ of 16.2 ± 4.5 nM (Figure 5C). The rise in intracellular Ca^2+^ concentration induced by 30 nM GSK1016790A was inhibited by RN1734 pretreatment (30 μM) a well-known TRPV4 antagonist (Figures 5D,E).

## Discussion

Brain microvessel endothelial cells, the main component of the BBB, are widely exposed to various stimuli from the blood and brain compartments. Among these, blood borne compounds and mechanical stress might modulate the activity of TRP channels, thus allowing Ca^2+^ entry into brain endothelial cells where Ca^2+^ concentrations need to be maintained within a certain range of concentrations through a dynamic equilibrium (Ma and Liu, 2019). Intracellular/cytosolic [Ca^2+^] modulates various cellular processes such as excitability, proliferation, synaptic plasticity, resistance to oxidative stress, and cell death, depending on the concentration, timing and duration of the signal (Fliniaux et al., 2018). Modifications of intracellular [Ca^2+^] can trigger the proliferation of BMEC and increase the permeability of the BBB by altering tight junction proteins (Ma and Liu, 2019). Furthermore, BBB dysfunctions observed in several brain pathologies are usually associated with increasing intracellular Ca^2+^ concentrations, suggesting that TRP-mediated Ca^2+^ signaling may play crucial roles in maintaining and regulating BBB functions (De Bock et al., 2013). Interestingly, the highly potent TRPV2 activator CBD increased Ca^2+^ entry in hCMEC/D3 cells where it induces proliferation, tubulogenesis and migration (Luo et al., 2019). Moreover, CBD has anti-inflammatory activity and is able to reduce the disruption of the BBB observed in lipopolysaccharide (LPS) (Ruiz-Valdepenas et al., 2011) and multiple sclerosis mice models (Mecha et al., 2013). CBD was demonstrated as effective in preventing disruption of an *in vitro* BBB model under hypoxic and glucose deprivation conditions (Hind et al., 2016). Altogether, these results suggest that activating TRPV2 might be a relevant strategy to positively modulate the BBB in the context of brain disorders including ischemic stroke (Calapai et al., 2020). Here, we looked at interspecies differences in the expression and activity of TRPV in rat and human BBB.

Transient receptor potential vanilloid 1-4 were first to be known for their expression in sensory neurons and their function in sensing nociception and pain (Mickle et al., 2015). However, the expression of TRPV1-4 in the brain is quite region-specific and heterogeneous (Kauer and Gibson, 2009; Cavanaugh et al., 2011; Nedungadi et al., 2012). For TRPV1, previous studies have believed that it can be widely expressed throughout the brain (Kauer and Gibson, 2009). However, a recent study applied a highly sensitive method using gene editing to modify the Trpv1 genetic locus in two lines of reporter mice, showing that TRPV1 is highly limitedly expressed in several specific brain regions, such as the caudal hypothalamus, the dorsal motor nucleus of the vagus, mesencephalic trigeminal nucleus, and parabrachial nucleus (Cavanaugh et al., 2011). Litter expression was found for the other brain regions (Cavanaugh et al., 2011). TRPV2 was found to be intensively expressed mainly in several brain areas including: hypothalamus, the nucleus of the solitary tract, hypoglossal nucleus, and the rostral division of the ventrolateral (Nedungadi et al., 2012). TRPV3 protein expression has not been detected in the mouse brain, although TRPV3 mRNA transcripts has been detected in the brain at low levels (Luo and Hu, 2014). TRPV3 is also evidenced to be absent in the mouse bEnd.3 brain endothelial cell line and in the freshly isolated brain capillaries and primary cultured BMECs from rat (Mickle et al., 2015). This is in accordance with the whole transcriptomic analysis demonstrating the absence of Trpv3 in murine primary BMECs (Zhang et al., 2014) and by brain vascular single-cell transcriptomics study (Vanlandewijck et al., 2018). TRPV3 is most abundantly expressed in skin keratinocytes and in cells surrounding hair follicles, where it plays an essential role in cutaneous sensation. RT-PCR or q-PCR analysis showing the absence of TRPV3 in human BMECs (Golech et al., 2004; Hatano et al., 2013) and gain-of-function mutations in human TRPV3 are associated with Olmsted syndrome, which is characterized by severe palmoplantar and periorificial keratoderma (Greco et al., 2020; Zhang et al., 2020). TRPV4 has been reported to be widely expressed throughout the brain including hippocampus, hypothalamus, cerebellum, lamina terminalis and optic chiasm, as well as a special enhanced expression in the olfactory placodes (Mangos et al., 2007; Kauer and Gibson, 2009; Shibasaki et al., 2015). Unfortunately, it is still remained largely unknown which types of cells were responsible for their expression in a specific brain region (Kauer and Gibson, 2009). Our results showed significant mRNA levels of some TRPVs in human brain endothelial cells which follow this rank of expression: *TRPV2* > *TRPV4* > *TRPV1*, while *TRPV3* was barely detected. It should be noted that this rank order is based on mRNA levels and functional studies since the absolute amount of each TRPV at protein level was not investigated in this study. These observations are supported by a study showing the qualitative expression of TRPV1, 2, and 4 in human primary cultured brain endothelial cells by RT-PCR (Hatano et al., 2013). TRPV2, a thermo-sensitive TRPV channel, was by far the most highly expressed isoform in hPBMEC and hCMEC/D3 while its relative expression was much lower than that of Trpv4 in rat microvessels and rPBMEC. TRPV1 and TRPV4 were also expressed both in rPBMEC, hPBMEC and hCMEC/D3 cells, and TRPV2 was even more expressed in hPBMEC than in hCMEC/D3 cells. TRPV4 expression was further evidenced by Western blot and immunofluorescence analysis in hCMEC/D3 cells supporting previous RT-PCR and/or immunofluorescence results in human BMEC (Sullivan et al., 2012; Hatano et al., 2013). However, using specific peptides of TRPV2 and TRPV4 targeted by the primary anti-TRPV2 and antiT-RPV4 antibodies used in our study would have increased the specificity of the immunofluorescence staining. Interestingly, we detected a 4.0- and 5.8-fold higher expression of, respectively, *TRPV1* and *TRPV4* mRNA, in the brain endothelial cells derived from the adult patient (CC216) as compared with the two children (CC205 and CC206), suggesting that TRPV1 and TRPV4 expression might increase with age while that of TRPV2 might decrease. This is in agreement with the reported age-dependent expression and function of Trpv1 in the rodent forebrain (Koles et al., 2013), although we cannot exclude, in our case, that these differences might result from the limited numbers of patients and/or reflect the underlying pathology or the exposure of the patients to medication. Interestingly, *TRPV* expression in hPBMEC was modulated by co-culture with astrocytes as shown by the 4.8-fold TRPV1 increase in hPBMECs co-culture with isolated primary astrocytes obtained from the same patient. In contrast, this effect was absent for *TRPV2,3,4* isoforms. Recently, Mannari et al. (2013) have reported that the astrocytic *TRPV1* could directly sense the blood-borne signals in the sensory circumventricular organs in adult mouse brains. Trpv1 is regarded as a target in regulating BBB permeability (Hu et al., 2005; Beggs et al., 2010) and might be also a downstream target in the modulated BBB permeability mediated by astrocytes (Abbott, 2002; Abbott et al., 2006).

A similar expression profile was found between hPBMEC isolated from patients and the human BBB cell line hCMEC/D3, confirming previous data (Dauchy et al., 2009; Ohtsuki et al., 2013) and suggesting that hCMEC/D3 is a proper *in vitro* surrogate in exploring TRPV properties at the human BBB. *Trpv2* gene expression was 25.8-fold more expressed in brain cortex than in rPBMEC, in accordance with a more pronounced Trpv2 immunostaining in the rat brain (Nedungadi et al., 2012). Notably, we evidenced a remarkable interspecies difference regarding the expression profile of *TRPV1-4* between rPBMEC and the two human *in vitro* BBB models (hPBMEC and hCMEC/D3). Although *TRPV2* and *TRPV4* were expressed both in rat and human BMEC, *Trpv4* and *TRPV2* were the most expressed isoform in rPBMEC and hBMEC, respectively. Brown et al. (2008) have demonstrated *Trpv1-4* expression by RT-PCR in the bEnd.3 murine BBB cell line and in isolated mouse brain microvessels, showing a higher *Trpv2* expression than *Trpv4*, suggesting TRPV expression profile could be similar in murine and human BMEC. A whole transcriptomic analysis revealed a similar expression of *Trpv2* and *Trpv4* in murine primary BMEC, while *Trpv1* and *Trpv3* transcripts were undetectable (Zhang et al., 2014). Except for TRPV1-4, many other transporters and enzymes expressed at the BBB also demonstrated interspecies differences in expression and function levels (Deo et al., 2013). PET studies have demonstrated a significant interspecies differences for the expression and functionality of ABCB1 (i.e., P-gp), an important efflux transporter at the BBB (Syvanen et al., 2009). The brain-to-plasma concentration ratio of [^11^C]GR205171, a substrate of P-gp, was almost 9-times higher in humans compared with rats (Syvanen et al., 2009; Ball et al., 2013). In fact, interspecies differences in expression and/or function levels were also verified for ABCG2 (BCRP) (Ito et al., 2011; Uchida et al., 2011; Agarwal et al., 2012), MRPs (Agarwal et al., 2012), some OATPs (Ito et al., 2011; Uchida et al., 2011) and some SLCs (Ito et al., 2011; Agarwal et al., 2012).

Our current work did not indicate whether TRPV receptors are expressed at BBB in a polarized way or not. Many receptors/transporters are localized specifically at luminal or abluminal side of the brain endothelial cells in charge of different functions (Dragoni and Turowski, 2018). Very poor data were reported regarding the subcellular localization of TRPV1-3 in brain microvessels. In the rat middle cerebral arteries, TRPV4 was found to be expressed preferentially on the abluminal membrane of endothelial cells (Marrelli et al., 2007) but the observation that a selective P2Y2 receptor agonist could induce TRPV4-mediated Ca^2+^ entry across the luminal and abluminal face of an endothelial monolayer (Guerra et al., 2018), suggests that TRPV4 could be also expressed on the luminal face of endothelial cells. Benfenati et al. (2007) confirmed that expression of TRPV4 is abundantly localized on astrocytic endfeet area abutting pial and parenchyma blood vessels. Also, TRPV2 has been shown to be prominently localized to the umbrella apical membrane of bladder epithelium cells, while TRPV4 was identified on their abluminal surfaces (Yu et al., 2011).

Transient receptor potential vanilloid-mediated modulation of the intracellular Ca^2+^ concentration was further investigated using specific agonist and antagonists. The highly potent TRPV1 agonist CAP, usually effective at nanomolar levels (Flockerzi, 2007), did not increase the AUC of intracellular Ca^2+^ during 20 min in hCMEC/D3 at a concentration of 1 or 3 μM. Further statistical tests revealed that 3 μM CAP can stimulate the Ca^2+^ influx at specific time (7, 11, and 12 min post CAP stimulation), indicating a possible weak function of TRPV1 in the BBB. This in agreement with the low gene expression of *TRPV1* in hCMEC/D3 and also with the recent observation that no functional activity with CAP up to 1 mM could be detected in human renal podocytes although these cells expressed TRPV1 (Ambrus et al., 2017). Notably, TRPV1-induced Ca^2+^ influx under CAP stimulation at specific times is not quite dose-dependently as similar Ca^2+^ influx levels were found in 1 or 3 μM CAP, from the view of either the AUC of Ca^2+^ during 20 min or the Ca^2+^ influx at any specific time, further identifying the quite lower TRPV1 expression at the membrane of hCMEC/D3 cells. In fact, the much lower expression of TRPV1 in BBB endothelial cells were also demonstrated by Golech et al. (2004) by RT-PCR and polyclonal antibodies. TRPV1 is also functional in responding to heat stimulation, however the heat-induced Ca^2+^ influx was not significantly inhibited by CPZ (10 or 20 μM), a widely used TRPV1 antagonist, suggesting that TRPV1 might not be the main functional isoform in hCMED/D3 cells. In addition, it was reported that TRPV1 could be also activated by CBD, although its agonist potency was lower than toward TRPV2 (Qin et al., 2008). Yet, no significant alteration in intracellular Ca^2+^ was observed when co-treating cells with CBD and CPZ, a TRPV1 antagonist, further suggesting the lack of TRPV1 activity or weak TRPV1 activity in hCMEC/D3 cells. However, we cannot exclude a functional activity of TRPV1 in hPBMECs since TRPV1 gene expression was much higher in those cells than in hCMEC/D3 cells, especially when they were co-cultured with astrocytes from the same patient. Moreover, CPZ is not a very potent antagonist of TRPV1 (Rigoni et al., 2003), that could only produce an insufficient/partial blocking of TRPV1. Therefore, the absence of functional activity of TRPV1 in hCMEC/D3 cells remains questionable.

The specific and potent TRPV4 agonist GSK triggered a rapid elevation of intracellular Ca^2+^ with an E_*max*_ at 100 nM (EC_50_ ∼16.2 nM), which can be totally inhibited by the specific TRPV4 antagonist RN1734. The EC_50_ for GSK in hCMEC/D3 cells was close to that reported for stimulating Ca^2+^ influx in another study (26.9 nM) (Sullivan et al., 2012). Previous studies have identified the expression of TRPV4 in commercial human BMECs at gene and protein levels (Hatano et al., 2013). The expression and function of TRPV4 are also verified using patch-clamp electrophysiology techniques in freshly BMECs isolated from brain capillaries of C57BL/6J mouse (Harraz et al., 2018). The specific TRPV4 agonist GSK-stimulated currents was completely abolished in TRPV4-knockout mice or when co-applying the specific TRPV4 antagonist HC-067047 in murine BMECs (Harraz et al., 2018). It is also reported that TRPV4 might play a vital role in regulating the function of blood-retina barrier, similarly to BBB, via modulating Ca^2+^ homeostasis in human retinal microvascular endothelial cells (Phuong et al., 2017). Due to their diverse cellular and subcellular localizations, TRP channels could mediate Ca^2+^ influx and generate distinct cytosolic/local intracellular Ca^2+^ waves that could regulate specific subcellular downstream pathways leading to different cell responses including death or survival (Fliniaux et al., 2018). In this regard, mitochondrial expression of TRPV1-2-3 has been evidenced in human odontoblasts (Wen et al., 2017), which is consistent with the intracellular staining we observed in hCMEC/D3 cells for TRPV2 and TRPV4.

## Conclusion

In conclusion, our present study shows that TRPV1, TRPV2 and TRPV4 are expressed in hPBMECs. Even less expressed than TRPV2, TRPV4 is functionally expressed in the human BBB. Recently, TRPV4 has been shown to be highly expressed and functional in retinal endothelial cells (Phuong et al., 2017). The authors nicely showed by different electrophysiological and Ca^2+^ imaging techniques and using an *in vitro* retinal barrier model that TRPV4 highly contributed to Ca^2+^ homeostasis and barrier function. The role of TRPV4 in BBB barrier function and physiology should be evaluated in further experiments before claiming whether or not TRPV4 could be a target to be investigated for modulating the BBB. TRPV1 is much less expressed than TRPV2 and TRPV4 in the human BBB but still shows weak or limited functionality when activated by relative high concentration of selective chemical agonist. An interspecies difference between rat and human TRPV2,4 was evidenced in the BBB using a gene expression approach which may be taken into consideration for rat to human translational pharmacological approaches targeting TRPV in the BBB. This interspecies difference between rat and human TRPV should be further confirmed from a functional point of view by using for example electrophysiological techniques like patch-clamp and measuring whole-cell currents of TRPV2 and TRPV4 between human and rat BMEC.

## Data Availability Statement

The datasets presented in this study can be found in online repositories. The names of the repository/repositories and accession number(s) can be found in the article/supplementary material.

## Ethics Statement

The studies involving human participants were reviewed and approved by French Ministry of Higher Education and Research (CODECOH DC-2014-2229). Written informed consent to participate in this study was provided by the participants’ legal guardian/next of kin. The animal study was reviewed and approved by the Ethics Review Committee of Paris Descartes University.

## Author Contributions

HL, BS, SCh, VC, MS, FG, and CC: experiments. HL and XD: writing – original draft preparation. BS, SCi, and XD: writing – review and editing. SCi and XD: supervision. All authors have read and agreed to the submitted version of the manuscript.

## Conflict of Interest

NP and FG were employed by the company BrainPlotting. The remaining authors declare that the research was conducted in the absence of any commercial or financial relationships that could be construed as a potential conflict of interest.
